# Frequency of LCT -13910C>T single nucleotide polymorphism associated with adult-type hypolactasia/lactase persistence among Brazilians of different ethnic groups

**DOI:** 10.1186/1475-2891-8-46

**Published:** 2009-10-02

**Authors:** Rejane Mattar, Maria S Monteiro, Cibele A Villares, Aníbal F Santos, Joyce MK Silva, Flair J Carrilho

**Affiliations:** 1 Department of Gastroenterology, University of São Paulo School of Medicine, São Paulo, SP, Brazil

## Abstract

**Background:**

Adult-type hypolactasia, the physiological decline of lactase some time after weaning, was previously associated with the LCT -13910C>T polymorphism worldwide except in Africa. Lactase non-persistence is the most common phenotype in humans, except in northwestern Europe with its long history of pastoralism and milking. We had previously shown association of LCT -13910C>T polymorphism with adult-type hypolactasia in Brazilians; thus, we assessed its frequency among different Brazilian ethnic groups.

**Methods:**

We investigated the ethnicity-related frequency of this polymorphism in 567 Brazilians [mean age, 42.1 ± 16.8 years; 157 (27.7%) men]; 399 (70.4%) White, 50 (8.8%) Black, 65 (11.5%) Brown, and 53 (9.3%) Japanese-Brazilian. DNA was extracted from leukocytes; LCT -13910C>T polymorphism was analyzed by PCR-restriction fragment length polymorphism.

**Results:**

Prevalence of the CC genotype associated with hypolactasia was similar (57%) among White and Brown groups; however, prevalence was higher among Blacks (80%) and those of Japanese descent (100%). Only 2 (4%) Blacks had TT genotype, and 8 (16%) had the CT genotype. Assuming an association between CC genotype and hypolactasia, and CT and TT genotypes with lactase persistence, 356 (62.8%) individuals had hypolactasia and 211 (37.2%) had lactase persistence. The White and Brown groups had the same hypolactasia prevalence (~57%); nevertheless, was 80% among Black individuals and 100% among Japanese-Brazilians (*P *< 0.01).

**Conclusion:**

The lactase persistence allele, LCT -13910T, was found in about 43% of both White and Brown and 20% of the Black Brazilians, but was absent among all Japanese Brazilians studied.

## Background

Genetically programmed down-regulation of the lactase gene (adult-type hypolactasia) is detectable in children from the second year of life, although the onset and extent are somewhat variable [1]. The colonic micro flora ferments undigested lactose in the intestinal lumen, producing hydrogen, carbon dioxide and methane, provoking gastrointestinal symptoms characterized by bloating, flatulence, abdominal pain and diarrhea [2].

Lactase non-persistence is the most common phenotype in humans, with frequencies around 65%, except in northwestern Europe with its long history of pastoralism and milking [3]. Enattah et al. (2002) identified a variant allele polymorphism LCT -13910C>T upstream from the lactase gene locus, associated with hypolactasia/lactase persistence in Finland and elsewhere [4]; an exception is Africa, with three identified single nucleotide polymorphisms: LCT -14010G>C, LCT -13915T>G, and LCT -13907C>G [5].

The DNA region of the LCT -13910C>T lactase persistence-non persistence variant functioned in vitro as a *cis *regulatory element capable of enhancing differential transcriptional activation of the lactase promoter that is consistent with a causative role in the mechanism of lactase persistence/non-persistence phenotypes in humans [6].

The LCT -13910C>T polymorphism has been associated with adult-type hypolactasia in Brazilians [7]; thus, we assessed its frequency among different Brazilian ethnic groups.

## Methods

The local Ethics Committee approved this study. Randomly selected asymptomatic and dyspeptic individuals were invited to participate and gave written informed consent. Most symptomatic participants did not associate their symptoms with milk consumption. Ethnic groups were classified according to ethnicity as White, Brown (of White and African-Brazilian descent), Black, and Japanese-Brazilian. A total of 567 individuals were included [mean age, 42.1 ± 16.8 years; 157 (27.7%) men]; 399 (70.4%) White, 50 (8.8%) Black, 65 (11.5%) Brown, and 53 (9.3%) Japanese-Brazilian.

### Polymerase Chain Reaction-Restriction fragment length polymorphism analysis

The technique was previously described [7]. Briefly, DNA was extracted from leukocytes by salting out [8]. The previously described primers, LAC-C-M-U 5' GCTGGCAATACAGATAAGATAATGGA- 3' (position 26611-26636) and LAC-C-L-2 5'-CTGCTTTGGTTGAAGCGAAGAT-3' (position 26790-26811, Accession number AY220757) [9], were used to amplify the region surrounding the LCT -13910C>T polymorphism. Mulcare et al. [9] introduced a base change in the penultimate base of the LAC-C-M-U primer (G instead of T) such that the PCR product would be cut by *Hinf*I when the LCT-13910T allele is present. PCR products were digested by *Hinf*I (0.25 U) at 37°C overnight. *Hinf*I digestion of PCR products of the LCT-13910T allele resulted in 177 bp and 24 bp fragments; digestion of the LCT-13910C allele generated a single fragment of 201 bp. Positive controls (PCR products of LCT-13910CT and TT genotypes) were included to ensure complete digestion. Digested PCR products were visualized on a 3% low melting point agarose gel stained by ethidium bromide. Samples showing a single band of 201 bp were classified as the LCT-13910CC genotype or a single band of 177 bp as the LCT-13910TT genotype; two bands of 201 bp and 177 bp represented the LCT-13910CT genotype.

SPSS version 15 was used for statistical analysis.

## Results

Among the 567 individuals, the prevalence of LCT-13910CC genotype varied among ethnic groups. The White and Brown groups had the same prevalence (~57%); Black individuals had prevalence of 80%; and among Japanese-Brazilians, the prevalence was 100%. Only 2 (4%) Blacks had LCT-13910TT genotype, and 8 (16%) had the CT genotype. The White and Brown groups had similar LCT-13910CT genotype prevalence (37.3% and 40%, respectively). The rare LCT-13910TT genotype (4.9%, 28/567) occurred more frequently in the White group (6%, 24/399) than the Brown (3.1%, 2/65) (Table 1). Gender and allele frequencies showed no association.

**Table 1:** Genotypes and hypolactasia/lactase persistence distribution among the different ethnic groups (%).

**Genotypes**	**White**	**Brown**	**Black**	**Japanese-Brazilian**	**Total**
CC (hypolactasia)	226 (56.6)	37 (56.9)	40 (80.0)	53 (100)	356 (62.8)

CT	149 (37.3)	26 (40.0)	8 (16.0)		183 (32.3)

TT	24 (6.0)	2 (3.1)	2 (4.0)		28 (4.9)

Lactase persistence (CT and TT)	173 (43.4)	28 (43.1)	10 (20)^a^	None ^a^	211 (37.2)

Total	399 (100)	65 (100)	50 (100)	53 (100)	567 (100)

^a ^*P *< 0.01

Assuming an association between LCT-13910CC genotype and hypolactasia, and LCT-13910CT and TT genotypes with lactase persistence, 356 (62.8%) individuals had hypolactasia and 211 (37.2%) had lactase persistence. The White and Brown groups had the same hypolactasia prevalence (~57%); nonetheless, Black individuals and Japanese-Brazilians had 80% and 100%, respectively (*P *< 0.01) (Table 1).

## Discussion

The multi-ethnic Brazilian population includes descendents of Europeans, Amerindians (native population at the European arrival in 1500), and Africans brought as slaves from 1550-1800. Mitochondrial DNA analysis indicates that African-Brazilians carry the relative ancestral contribution of central-west, southeast, and west Africa [10], where the LCT-13910T variant of lactase persistence of Europeans in Africa was present [5]. Black-Brazilians have the LCT-13910T variant of lactase persistence although at a low frequency. However, most likely the LCT-13910T allele of European origin is the outcome of genetic admixture, a consequence of extensive interbreeding with Europeans that occurred through the centuries [10].

Brown and White individuals followed the same pattern of allelic frequencies. Interestingly, all Japanese-Brazilians had LCT-13910CC genotype, suggesting very low interbreeding between Japanese-Brazilian and European-Brazilian groups; nonetheless, these values agree with previous results of lactose malabsorption in White, and Black Brazilians and those of Japanese descent [11].

We conclude that the lactase persistence allele, LCT -13910T, was found in about 43% of both White and Brown and 20% of the Black Brazilians, but was absent among all Japanese- Brazilians studied.

## Competing interests

The authors declare that they have no competing interests.

## Authors' contributions

RM participated in the conception, design, analysis and interpretation of data, performed the statistical analysis, manuscript preparation and revision; MSM, CAV, AFS, JMKS carried out the molecular genetic studies and acquisition of data; FJC gave final approval of the version to be published. All authors read and approved the final manuscript.
